# Mature oligodendrocytes actively increase *in vivo* cytoskeletal plasticity following CNS damage

**DOI:** 10.1186/s12974-015-0271-2

**Published:** 2015-04-02

**Authors:** Giuseppe Locatelli, Arianna Baggiolini, Bettina Schreiner, Pushpalatha Palle, Ari Waisman, Burkhard Becher, Thorsten Buch

**Affiliations:** Institute of Experimental Immunology, University of Zurich, Winterthurerstrasse 190, Zürich, 8057 Switzerland; Institute of Clinical Neuroimmunology, LMU Universität München, Marchioninistrasse 17, Munich, 81377 Germany; Institute for Molecular Medicine, University Medical Center of the Johannes Gutenberg, University of Mainz, Obere Zahlbacher Str. 67, Mainz, 55131 Germany; Institute for Medical Microbiology, Immunology and Hygiene, Technische Universität München, Trogerstrasse 30, 80675 Munich, Germany; Institute of Laboratory Animal Science, VetSuisse, University of Zurich, Winterthurerstrasse 190, Zurich, 8057 Switzerland

**Keywords:** Demyelination, Experimental autoimmune encephalomyelitis, *In vivo* imaging, CNS plasticity, Cytoskeletal dynamics

## Abstract

**Background:**

Oligodendrocytes are myelinating cells of the central nervous system which support functionally, structurally, and metabolically neurons. Mature oligodendrocytes are generally believed to be mere targets of destruction in the context of neuroinflammation and tissue damage, but their real degree of *in vivo* plasticity has become a matter of debate. We thus investigated the *in vivo* dynamic, actin-related response of these cells under different kinds of demyelinating stress.

**Methods:**

We used a novel mouse model (oLucR) expressing luciferase in myelin oligodendrocyte glycoprotein-positive oligodendrocytes under the control of a β-actin promoter. Activity of this promoter served as surrogate for dynamics of the cytoskeleton gene transcription through recording of *in vivo* bioluminescence following diphtheria toxin-induced oligodendrocyte death and autoimmune demyelination. Cytoskeletal gene expression was quantified from mature oligodendrocytes directly isolated from transgenic animals through cell sorting.

**Results:**

Experimental demyelinating setups augmented oligodendrocyte-specific *in vivo* bioluminescence. These changes in luciferase signal were confirmed by further *ex vivo* analysis of the central nervous system tissue from oLucR mice. Increase in bioluminescence upon autoimmune inflammation was parallel to an oligodendrocyte-specific increased transcription of β-tubulin.

**Conclusions:**

Mature oligodendrocytes acutely increase their cytoskeletal plasticity *in vivo* during demyelination. They are therefore not passive players under demyelinating conditions but can rather react dynamically to external insults.

## Background

Oligodendrocytes (ODCs) are cells of the central nervous system (CNS) whose processes form myelin, a multi-layered membrane structure participating in saltatory signal conduction [1] and metabolic support of neuronal axons [2,3]. Myelin is produced in the last developmental stage of ODCs through a rapid, tightly regulated process [4] in which overlaying contiguous membranes become strongly interconnected by extruding cytoplasm to form compact myelin [5]. These membrane domains remain directly connected to the cell body with a complex underlying cytoarchitecture comprising microtubules distributed in larger processes and actin filaments enriched in thinner myelin domains and in paranodes [6,7]. β-actin and β-tubulin are thus main players in dynamics of axon targeting and myelin stability [7,8].

Acute or chronic damage to ODCs inevitably leads to neuronal loss as observed in several animal models [9-11] and human diseases such as multiple sclerosis (MS) and inherited leukodystrophies of the CNS [10]. However, demyelination and ODC death also lead to the activation of oligodendrocyte progenitor cells (OPCs) [11-15]. These cells can develop into mature ODCs and remyelinate naked axons, thus restoring saltatory conduction [16]. In this context, the role of surviving mature ODCs within and surrounding damaged CNS areas is still unclear. While it is current dogma that mature ODCs lack the ability to remyelinate axons [13,17], some studies indicate that these cells can at least maintain different degrees of structural plasticity. Earlier observations in different experimental paradigms and within MS lesions indicate sparse mature ODC proliferation within remyelinating areas [18-20], and *in vitro* ODCs can survive complement attack by actively shedding myelin vesicles [21], regenerate myelin processes after damage [22,23], and display migratory capability after maturation [24]. Also, the fact that ODCs close to or within neuroinflammatory lesions that have been deprived of their myelin processes can survive this insult [25,26] suggests the existence of active mechanisms of cellular plasticity.

Insights into dynamic properties of ODCs could come from the study of the cell cytoarchitecture which regulates and drives membrane movements [27]. In order to investigate the plasticity of mature ODCs under demyelinating conditions *in vivo*, we have thus generated a mouse model (oLucR) in which luciferase expression is controlled by a β-actin promoter and restricted specifically to mature ODCs [28]. CNS-specific bioluminescence in oLucR mice was quantitatively measured *in vivo* after injection of luciferin. We followed bioluminescence changes in two experimental models of ODC damage, namely diphtheria toxin (DTx)-mediated ODC killing (oDTR model [11,29,30]), and in the neuroinflammatory paradigm experimental autoimmune encephalomyelitis (EAE) [1]. oLucR mice revealed defined and reproducible increases in the *in vivo* bioluminescence during induced demyelination in both experimental paradigms, independently from *de novo* ODC generation from progenitors. The measured *in vitro* and *ex vivo* bioluminescence correlated with the *in vivo* longitudinal data, indicating that our observations revealed an intrinsic feature of the damaged ODC population. Transcriptional analysis of structural genes in the damaged CNS and specifically within ODCs showed increased expression of cytoskeleton genes after demyelinating insult. Our results thus elaborate in a novel *in vivo* model previous suggestions that ODCs undergoing/sensing cellular stress can transiently enhance their own plasticity [21-24]; furthermore, we provide important insights on the timing and extent of such activation in *in vivo* experimental demyelination models.

## Methods

### Animals

Mice were kept under SPF conditions according to Swiss and German animal laws and institutional guidelines. Animal experiments were conducted under the license numbers 13/2006 and 55.2-1-54-2532-1-12 after approval by the respective Swiss and German government agencies, the *Veterinäramt* of the Canton of Zurich and the *Regierung von Oberbayern*. The presence of respective transgenes was confirmed by PCR analysis on DNA from tail biopsies by the use of the following primer pairs: *MOGi-cre* [29] (WT 350 bp) GAC AAT TCA GAG TGA TAG GAC CAG GGT ATC CC and GCT GCC TAT TAT TGG TAA GAG TGG; *MOGi-cre* (knock-in, 700 bp) TCC AAT TTA CTG ACC GTA CAC and CAT CAG CTA CAC CAG AGA CGG AAA TC; *iDTR* [30] (WT 600 bp, KI 845 bp) AAA GTC GCT CTG AGT TGT TAT, GGA GCG GGA GAA ATG GAT AAA GTC GCT CTG AGT TGT TAT, GGA GCG GGA GAA ATG GAT ATG, and AAT AGG AAC TTC GTC GAG AAT AGG AAC TTC GTC GAG C; *Luciferase* (415 bp) TGT TGT TCC ATT CCA TCA CGG and ATC CAG ATC CAC AAC CTT CGC; and *EYFP* (200 bp) CTA TAT CAT GGC CGA CAA GC and ACT GGG TGC TCA GGT AGT GG.

#### In vivo *bioluminescence recordings and disease models*

oLucR mice were shaved and anesthetized, and light emission was recorded *in vivo* in an ultrasensitive IVIS 100 system (Caliper Life Science, Hopkinton, MA, USA) after intraperitoneal (i.p.) injection of 150 ng/kg of D-luciferin. The average photon-*per*-second recording over the full kinetics of the luciferase reaction was used as readout *per* mouse, *per* measurement.

For EAE experiments, 6- to 10-week-old mice were immunized subcutaneously with 200 μg (each flank 100 μg) of myelin oligodendrocyte glycoprotein MOG_35–55_ peptide (MEVGWYRSPFSRVVHLYRNGK) emulsified in Complete Freund’s Adjuvant (CFA; H37 Ra, Difco Laboratories, Franklin Lakes, NJ, USA), and injected i.p. the same day and at day 2 with 200 ng pertussis toxin (Sigma-Aldrich, St. Louis, MO, USA). To deplete ODCs through DTx administration, 6- to 10-week-old oLucR/diphtheria toxin receptor (DTR) animals were injected i.p. with 200 ng DTx (Merck, Darmstadt, Germany) daily over 7 days. EAE scoring and composite score of DTx-induced clinical disease was performed as in [11].

#### Ex vivo *CNS slices*

Animals were killed and perfused with phosphate-buffered saline (PBS), and CNS were collected and cut in half. From one half of the brain and spinal cord, approximately 1-mm-thick slices were cut, bathed in 150 μg/ml luciferin, and immediately recorded for 2 min within an IVIS 100 system (Caliper Life Science).

### Luminometer assay

Mouse tissue was dissolved in lysis buffer (Promega Corporation, Fitchburg, WI, USA). Protein content was quantified by the Bradford method (Bio-Rad, Munich, Germany) using BSA as protein standard. Lysates were adjusted to same protein content and analyzed according to manufacturer’s guidelines in a black 96-well plate on a microplate luminometer (Victor2, Wallac, Freiburg, Germany).

### Immunostaining of cells after cytospin

Cell sorting was carried out using a FACSAria III (BD Biosciences, San Jose, CA, USA). Dead cells were excluded using an Aqua Live/Dead staining reagent (Invitrogen, Carlsbad, CA, USA) and microglial/myeloid cells by positive staining for CD45 and CD11b. Sorted EYFP^+^ ODCs were spun onto glass slides (600 rpm) (Shandon Southern Instruments, Sewickley, PA, USA). Cells were fixed in 4% paraformaldehyde (10 min), incubated with 4% normal goat serum in 0.2% Triton-PBS, and then immunostained. For primary antibodies, goat anti-GFP antibody (Rockland, Gilbertsville, PA, USA), mouse anti-CC1 (Calbiochem, Darmstadt, Germany), rabbit anti-PLP (Abcam, Cambridge, UK), and Isolectin b4-Alexa Fluor 568 (Sigma-Aldrich, St. Louis, MO, USA) were used as indicated and were detected with secondary anti-goat Alexa Fluor 488-, anti-mouse Alexa Fluor 405-, anti-rabbit Alexa Fluor 647-conjugated antibody (all by Invitrogen, Carlsbad, CA, USA). Confocal microscopy was performed using a SP5 confocal laser microscopy (Leica, Wetzlar, Germany) and images analyzed using Imaris software (version 7.5.1; Bitplane, Zurich, Switzerland).

### Expression analysis

Samples were homogenized in 1 ml TRIZOL and incubated for 5 min at room temperature (RT). Two hundred microliters chloroform were added, incubated for 3 min at RT, and centrifuged at 8,000 rpm for 15 min at 4°C. The RNA in the aqueous phase was precipitated by 500 μl isopropyl alcohol (10-min incubation at RT and centrifugation at 800 rpm for 10 min at 4°C). The RNA pellet was washed with 1 ml 75% ethanol. Samples were centrifuged at 5,000 rpm for 5 min at 4°C, and the RNA pellet was dried for 15 min at RT and dissolved in 45 μl RNase free water with 5 μl of 10× incubation buffer and 1 μl DNaseI. The samples were incubated at 37°C for 20 min, and then, 1 μl 0.5 M EDTA was added, incubated at 75°C for 10 min, and placed on ice. For FACS-sorted cell samples, RNA was prepared using an RNeasy Plus Micro Kit (Qiagen, Valencia, CA, USA). One microliter random primers (100 ng/μl), 1 μl dNTP (10 mM), 4 μl first-strand buffer (5×), 2 μl DTT (0.1 M), and 1 μl RNase OUT were incubated for 2 min at 37°C, and then, 1 μl M-MLV RT (200 U/μl) was added.

Five micrograms RNA were diluted in 10 μl RNase free H_2_O, heated to 65°C for 5 min, and quenched on ice. Afterwards, 10 μl Master Mix were added to the RNA, incubated 10 min at 21°C, 50 min at 37°C, and finally 15 min at 70°C. The cDNA was finally diluted 1:10. Five microliters of cDNA per real-time reaction were mixed with 12.5 μl SYBR Green, 6.5 μl H_2_O, 0.5 μl F-Primer, and 0.5 μl R-Primer for a total volume of 25 μl. The real-time (RT-PCR) was performed using a C1000 Touch Thermal Cycler (Bio-Rad CFX384 Real-Time System, Bio-Rad, Munich, Germany) with the following primers: *β-actin*, F: AGA GGG AAA TCG TGC GTG AC; R: CAA TAG TGA TGA CCT GGC CGT; *Nestin*, F: CAA GAA CCA CTG GGG TC, R: CCC TCC TGG TGA TTC CAC A; *NG2*, F: GTT GGG ATG CTT GCT GG, R: TGA AAG CTG CAG AAG CA; *MOG*, F: AAA TGG CAA GGA CCA AG, R: AGC AGG TGT AGC CTC CTT; *OLIG1*, F: ACC AAC GTT TGA GCT TGC TT, R: GGT TAA GGA CCA GCC TGT GA; *β-Tubulin*, F: TCG TGG AAT GGA TCC CCA AC; R: CTC CAT CTC GTC CAT GCC CT; and *RNA polymerase II*: CTG GTC CTT CGA ATC CGC ATC and GCT CGA TAC CCT GCA GGG TCA.

### Immunohistochemistry

Mice were euthanized with CO_2_ and perfused with PBS. For cryostat sections, the tissue was fixed overnight with 4% PFA, cryoprotected in 30% sucrose and frozen at −80°C. Frozen tissue was cut sagittally in 40-μm-thick sections and stained with myelin basic protein (MBP)-specific (Dako, Glostrup, Denmark) and β-actin-specific (Biolegend, San Diego, CA) antibodies. Detection was accomplished using Alexa fluor-coupled secondary antibodies (Life Technologies, Karlsruhe, Germany). Sections were covered with Vectashield (Vector Laboratories, Burlingame, CA) and analyzed by confocal (Leica SP7, Leica, Wetzlar, Germany) microscope.

### Statistical analysis

Unless otherwise indicated, data were displayed as mean ± SEM, and statistical analysis performed by two-tailed Student’s *t* test using Excel software (Microsoft). Disease onset/bioluminescence increase correlation analysis was performed by Pearson’s correlation coefficient analysis.

## Results

### *In vivo* bioluminescence in the oLucR mouse model is specific for mature ODC

**Figure 1:**
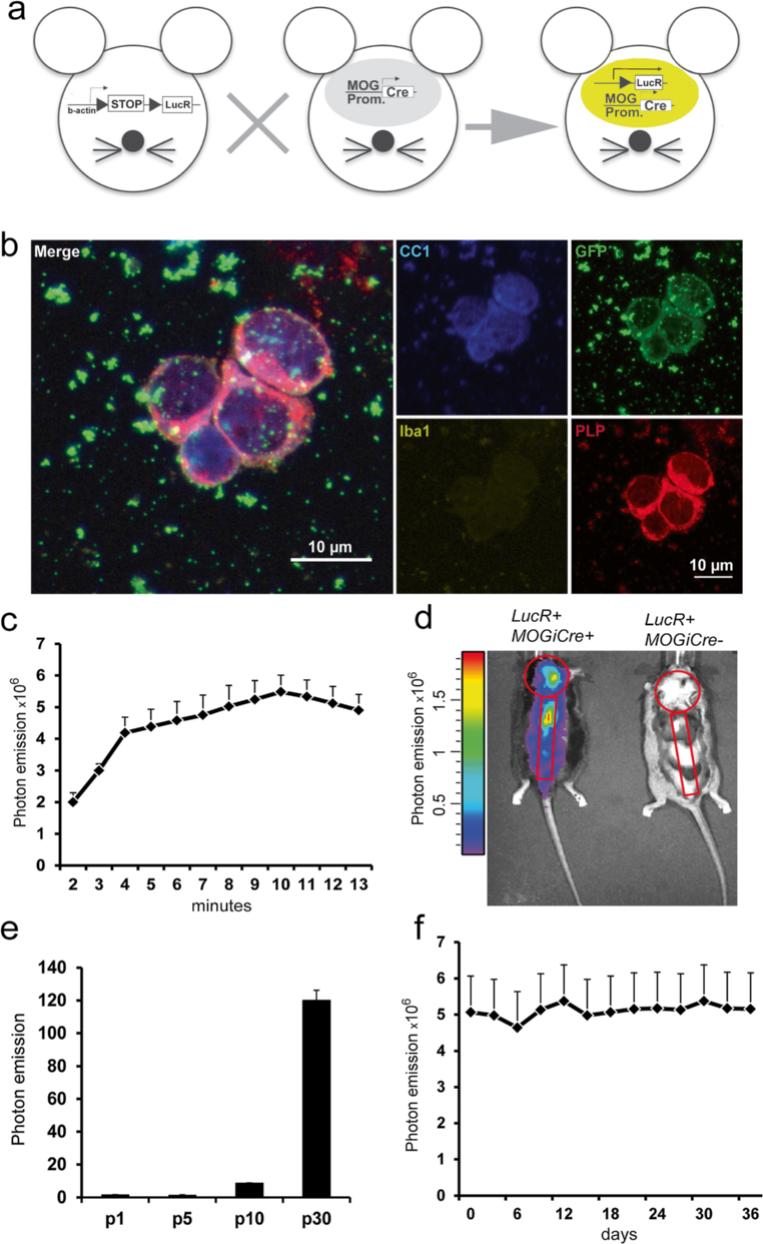
**The oLucR mouse model shows CNS-specific** ***in vivo*** **bioluminescence. (a)** ODC-specific expression of luciferase is achieved by crossing a Cre-inducible luciferase reporter mouse (left) to the MOGi-cre strain. **(b)** EYFP^+^ cells were sorted and stained with CC1-, GFP-, Iba1-, and PLP-specific antibodies following cytospin. Microglia/macrophage cells were excluded by positive CD45 and CD11b staining. Dead cells were excluded by Aqua Live/Dead staining reagent (Life Technologies). **(c)** Kinetics of photon emission acquired with an IVIS camera in anesthetized oLucR animals following intraperitoneal injection of 150 ng/kg of D-luciferin (mean ± SEM, *n* = 8). **(d)** *In vivo* bioluminescence recorded in a representative oLucR mouse (left) and a control LucR animal where the STOP codon impedes luciferase expression (right). Shown in red are the specific regions of interest (ROIs) for signal acquisition. **(e)** The CNS from oLucR mice of the indicated ages were homogenized and analyzed in a luminometer assay. Photon emission of the lysates is shown (mean ± SEM, *n* = 3). **(f)** oLucR mice were injected with luciferin every 3 days and bioluminescence recorded from specific brain and spinal cord ROIs over the course of 36 days (mean ± SEM, *n* = 4).

The aim of our study was to investigate cellular dynamics of ODCs in an *in vivo* mouse model. As microfilament dynamics control the fine morphological changes during OPC migration, differentiation, and ODC myelination, the activation of the β-actin promoter was used as indirect readout of ODC plasticity. We generated a mouse model (oLucR) in which activity of the β-actin promoter can be monitored specifically within ODCs through luciferase expression. We achieved this by using a strain in which luciferase was driven by the β-actin promoter but its expression restricted to ODCs through excision of a loxP-flanked STOP cassette by the MOGi-cre strain specific for mature ODCs [28,29] (Figure 1a). Specificity and efficacy of the Cre-mediated recombination for terminally differentiated ODCs were tested by breeding the MOGi-cre mouse line with the EYFP reporter line, in which expression of the fluorescent protein EYFP was dependent on excision of a STOP cassette. Between 65% and 95% of CC1^+^, ODCs showed efficient locus recombination, depending on the CNS region (data not shown). To further characterize these cells, we FACS-sorted EYFP^+^ cells from dissociated CNS tissue of MOGi-cre x EYFP reporter mice. FACS-sorted ODCs appeared devoid of myelinated processes but remained alive (see ‘Methods’ section) and stained positive for PLP (myelin proteolipid protein) and CC1, both markers of mature ODCs (Figure 1b). Also, RT-PCR of sorted EYFP^+^ cells revealed strong expression of the mature ODC marker gene MOG and no expression of the precursor markers NG2 or nestin (data not shown). We could thus confirm *ex vivo* that mature ODCs physically deprived of their myelin sheaths can indeed survive the insult [23,25,26]. Taken together, Cre activity in MOGi-cre mice was confirmed to be restricted to mature ODC.

*In vivo* bioluminescence in oLucR mice was visualized and quantified in an IVIS 100 system (Caliper Life Science) after i.p. injection of 150 ng/kg of luciferin. The luciferase signal was characterized by reproducible kinetics of photon emission showing an emission peak 10 min after luciferin injection (Figure 1c). Since minor photon emission was also detected from non-CNS areas (such as liver, tail, and paws), we recorded the luciferase signal from specific regions of interest (ROIs) covering the brain and spinal cord (Figure 1d). While faint bioluminescence signals from the liver and spleen were expected [28], photon emission from the tail and paws proved to be an *in vivo* artifact, as freshly dissected tails and limbs did not show any luciferase expression *ex vivo* (data not shown). As expected, absolute luciferase content in the CNS of oLucR mice increased during postnatal development reflecting ODC maturation and thereby MOG expression [31] (Figure 1e). Longitudinal analysis of the luciferase signal in adult oLucR mice showed stable luciferase-driven bioluminescence over extended time periods (up to 5 weeks), as a consequence of steady-state expression of β-actin gene in undisturbed myelin (Figure 1f). Taken together, we showed that the oLucR system was suitable to indirectly visualize the *in vivo* activity of the β-actin promoter within mature ODCs.

### DTx-mediated ODC death increases overall ODC β-actin promoter activity

ODC death and demyelination are pathological hallmarks of several human CNS diseases such as certain hereditary and metabolic leukodystrophies and MS [1,32]. While it has been shown in several animal models that remyelination by differentiating OPCs follows myelin destruction [16], the behavior of the surviving mature ODC population in response to demyelinating stress or even death of nearby ODCs is still unclear. To investigate dynamics of the cytoskeleton in mature ODCs after demyelinating insults, we used the oDTR system [11] for experimental ODC death. We crossed oLucR mice to the iDTR strain [30] to obtain DTR expression specifically on MOG^+^ ODC. We recently showed that in the resulting oDTR system, DTx administration leads to a highly specific ablation of up to 60% of ODCs in disseminated white matter areas, followed by diffuse demyelination starting 1 week post administration (p.a.) [11]. In this model, surviving unaffected mature ODCs are often located scattered within demyelinated areas or in close proximity to damaged ODC neighbors [11].

**Figure 2:**
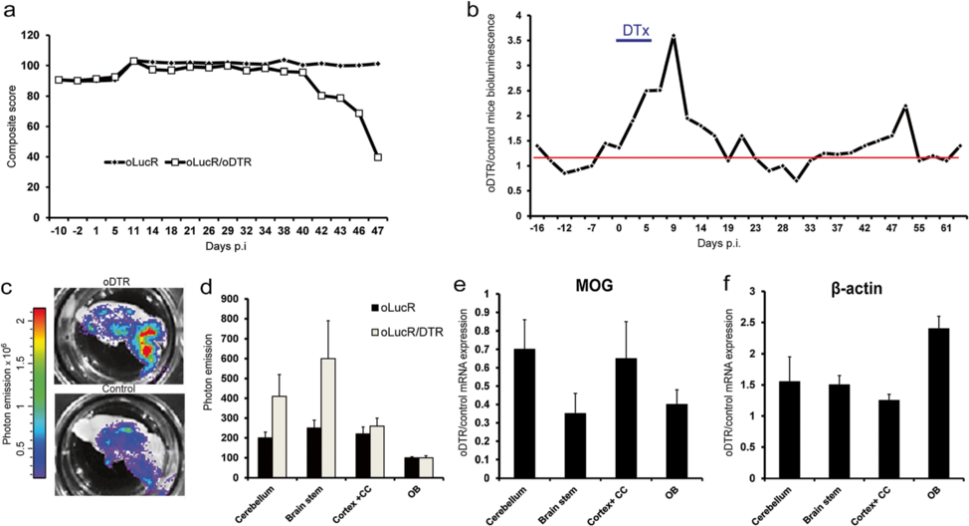
**Toxin-induced ODC death and demyelination leads to increased bioluminescence in oLucR mice. (a)** Composite score of clinical disability in DTx-treated oLucR/DTR and control oLucR mice (*n* = 5). Details of score in [11]. **(b)** Ratio of mean CNS-specific bioluminescence between DTx-injected oLucR/DTR and oLucR control mice imaged in an IVIS three times a week. Data are representative of two independent experiments (*n* = 10). The red line indicates the mean baseline photon emission before DTx treatment. The blue bar indicates the period of DTx treatment. **(c)** Representative pictures of freshly prepared luciferin-bathed brain slices from DTx-treated oLucR/DTR and oLucR control mice 7 weeks p.a. An overlay of photographic picture and photon emission is shown. **(d)** CNS from DTx-treated oLucR/DTR and control oLucR mice 7 weeks p.a. were dissected, and lysates were analyzed by luminometer (mean ± SEM, *n* = 4). Gene expression changes in the CNS of oDTR and control mice following DTx treatment. mRNA levels of MOG **(e)**, and β-actin **(f)** were measured in oLucR/DTR and control mice 9 days after DTx injections (*n* = 4, mean ± SEM). OB = olfactory bulb; CC = corpus callosum.

We injected into these oLucR/oDTR and into control oLucR mice 200 ng of DTx daily for a week. As a consequence of damage to the ODC population, DTx-treated oLucR/oDTR mice, but not oLucR control mice, presented with progressive and severe motor impairment starting 5 weeks p.a. (Figure 2a). In oLucR/oDTR mice, bioluminescence increased approximately threefold compared to the basal level during the first week of DTx administration (Figure 2b), parallel to the initial demyelination classically observed in the oDTR model [11]. To confirm that this signal peak was not the result of a damaged blood brain barrier (BBB) and thus easier access of luciferin to the brain parenchyma, we performed additional analyses. First, freshly prepared brain slices were placed in luciferin followed by *ex vivo* analysis of light emission (Figure 2c); second, total brain lysates were assessed by luminometer measurements at the end of the demyelination period (Figure 2d). Both assays confirmed increased luciferase expression in the CNS of DTx-treated oLucR/oDTR mice compared to controls. Furthermore, to support our observation obtained with the transgenic luciferase reporter system, we analyzed β-actin RNA levels in samples from different CNS areas. Since β-actin was the gene of interest in our analysis, we utilized transcript level of RNA polymerase II as housekeeping gene and internal control in this and following assays [33]. We found that, in contrast to the decrease in MOG expression (Figure 2e), β-actin was upregulated in all CNS regions during the initial period of increased *in vivo* luciferase activity (Figure 2f). Taken together, sterile ODC ablation led to increased ODC-specific, β-actin-driven bioluminescence, thus suggesting activation of cytoskeletal plasticity in ODCs upon demyelinating stressors.

### Increased activity of β-actin-driven luciferase at onset of autoimmune CNS inflammation

**Figure 3:**
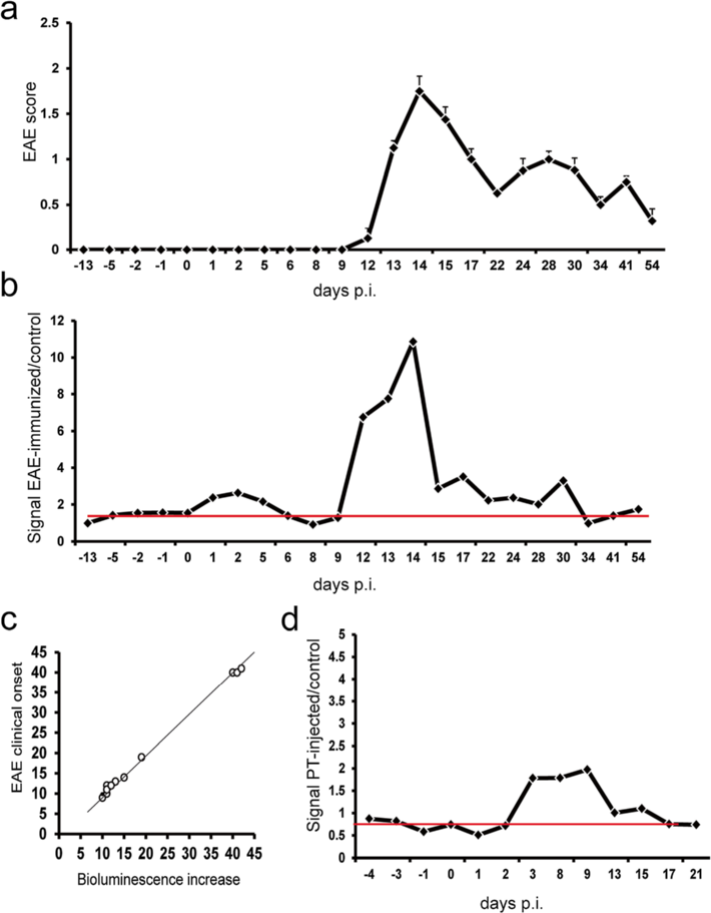
**CNS autoimmune inflammation leads to increased bioluminescence in oLucR mice at clinical onset of disease. (a)** EAE disease scores in MOG-immunized oLucR animals (mean ± SEM, *n* = 5). **(b)** Ratio of mean CNS-specific bioluminescence between MOG-immunized and control mice imaged in an IVIS every 2 to 3 days after immunization. Data are representative of three independent experiments (*n* = 15). The red line indicates the baseline photon emission before MOG immunization. **(c)** Linear correlation between day of clinical onset and increase of bioluminescence in MOG-immunized oLucR mice (*n* = 15, Pearson’s correlation coefficient = 0.9989). The three mice with disease onset around day 40 did not develop clinically overt EAE after the initial EAE induction and therefore were re-immunized with MOG peptide after 1 month. **(d)** Ratio of mean CNS-specific bioluminescence between PT-treated and control mice. oLucR mice were injected with 300 ng PT at day 0 and 2 and bioluminescence acquired over time (*n* = 5). The red line indicates baseline photon emission before PT administration.

**Figure 4:**
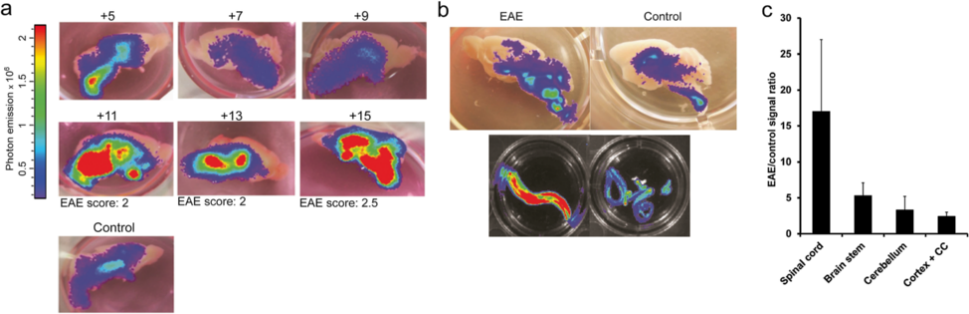
***Ex vivo*** **and** ***in vitro*** **increase in CNS-specific bioluminescence during neuroinflammation. (a)** Representative freshly dissected luciferin-bathed brain slices from oLucR animals at different time points following MOG immunization. An overlay of photographic picture and photon emission is shown. The EAE score at the time of analysis is shown below the individual pictures. **(b)** Representative freshly dissociated luciferin-bathed brain slices and whole spinal cords from MOG-immunized animals and not immunized control mice. Overlay of photographic picture and photon emission is shown. **(c)** CNS from MOG-immunized and control oLucR mice 25 days p.i. were dissected, and luciferase activity within lysates was analyzed by luminometer (mean ± SEM, *n* = 4).

**Figure 5:**
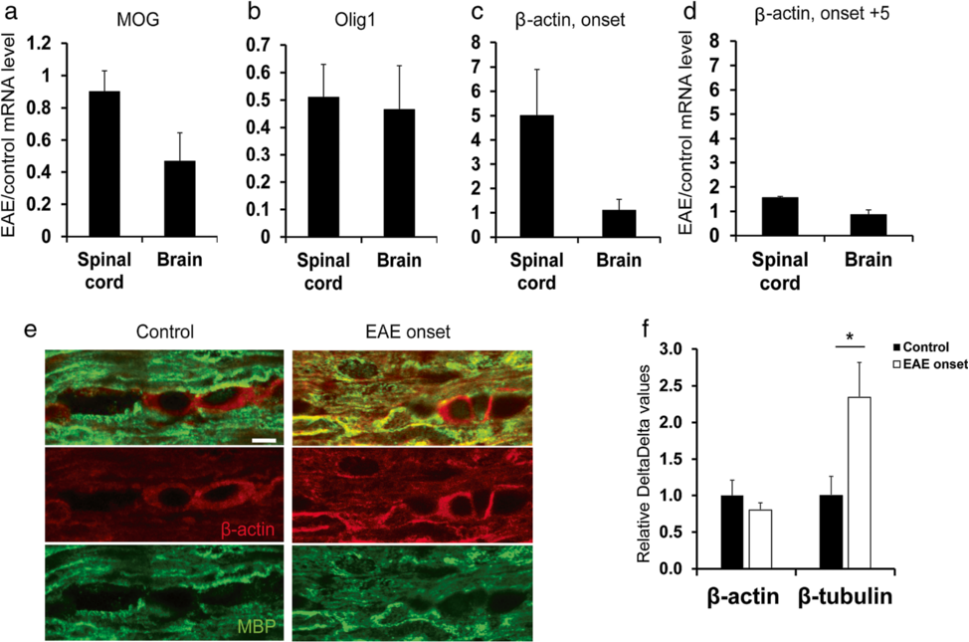
**RT-PCR analysis in the CNS of MOG-immunized versus control mice reveal upregulated expression of cytoskeleton genes.** mRNA levels of MOG **(a)**, NG2 **(b)**, and β-actin **(c)** were measured in MOG-immunized and not immunized (control) oLucR mice at clinical onset of EAE **(d)** or at onset +5 (mean of EAE/control ratio ± SEM, *n* = 5). **(e)** Sagittal sections of spinal cord from mice at disease onset and controls were immunostained with MBP- and actin-specific antibodies. Shown are representative pictures of ODCs in white matter areas (*n* = 3). Scale bar, 6 μm. **(f)** Relative mRNA expression in FACS-sorted EYFP^+^ ODCs from the CNS of MOG peptide immunized mice and naïve controls at day of clinical onset of EAE (mean ± SEM, *n* = 5). Two-tailed Student’s *t* test, *P < 0.05.

DTx-mediated pathology derives from direct, sterile insults to ODCs [11]. Yet, the most common demyelinating disease in humans is MS, in which an inflammatory process results in demyelination and axonal degeneration [34]. To assess whether immune-mediated damage to the CNS would induce an active plastic reaction of the mature ODC compartment, we measured the β-actin-driven bioluminescence response in oLucR mice in the EAE model. Mice were immunized against a myelin-derived peptide (MOG_35–55_), leading to a T cell-mediated attack against CNS myelin and to direct and indirect insults to ODCs [9]. In this system, a variable degree of demyelination and cell death is observed, predominantly in the spinal cord and brain stem [1]. We thus recorded bioluminescence every 2 to 3 days in immunized and in naïve control (oLucR) animals. After a transient increase in bioluminescence at day 2 post immunization (p.i.), we detected a sharp, approximately 12-fold increase in luciferase signal compared to baseline (Figure 3a,b). Such bioluminescence increase was observed consistently at the disease onset in MOG_35–55_-immunized oLucR mice (Figure 3a,b) and was followed by a sharp decrease in bioluminescence almost reaching levels found before immunization and in naïve animals (Figure 3b). Notably, the acute peak in bioluminescence consistently correlated with the beginning of clinical EAE signs independent of the exact time point of disease onset (Figure 3c). At the same time, bioluminescence in MOG-immunized oLucR mice that did not develop overt clinical paralysis remained unchanged over time (data not shown). This also indicated that not MOG immunization *per se*, but injury to ODCs and subsequent neurological deficits were closely related to actin/cytoskeleton ODC dynamics. We then tested whether increase in bioluminescence could have been the result of higher access of luciferin to the CNS. First, we investigated whether the adjuvant pertussis toxin (PT) alone, known to transiently increase BBB permeability [35,36], could affect *in vivo* bioluminescence in our model. PT administration in oLucR animals resulted in a low and transient increase in luciferase signal (Figure 3d), similar to the minor bioluminescence increase observed in the EAE experiment 1 to 5 days post immunization (Figure 3b). Second, we immunized oLucR animals with MOG_35–55_ in CFA and analyzed luciferin-bathed brain slices every other day from day 5 to day 15 p.i. *Ex vivo* bioluminescence followed similar dynamics as the one observed *in vivo,* with increased luciferase signals detected in all CNS regions of mice developing clinical EAE, albeit predominantly in the severely affected spinal cord (Figure 4a,b). In contrast, CNS slices from immunized mice that were found not to develop the disease did not show any increase in *ex vivo* bioluminescence (data not shown). Third, *in vitro* analysis of luciferase activity of CNS lysates revealed increased reporter levels especially in the spinal cord and brain stem of EAE-induced animals compared to controls (Figure 4c). Thus, intrinsic damage-related dynamical responses of the cytoskeleton within the ODC population account for the major bioluminescence changes observed in oLucR mice. To further investigate the nature of the bioluminescence increase at EAE onset, we immunized oLucR animals and analyzed gene expression in the spinal cord at day 11 p.i. We observed a decrease in ODC markers such as MOG and Olig1 (Figure 5a,b), while β-actin levels were strongly increased specifically in oLucR animals induced with EAE (Figure 5c). Notably, 5 days after clinical EAE onset, at the beginning of the ameliorating remitting phase, β-actin expression decreased almost reaching control levels (Figure 5d). As several invading immune cells and resident activated glial cells might contribute to the observed dynamics in whole-tissue β-actin transcription, we FACS-isolated mature EYFP^+^ ODCs from naïve and MOG-immunized EYFP reporter mice and analyzed expression level of cytoskeletal genes within these cells. In this assay, β-actin levels appeared unchanged in ODCs during the course of EAE (Figure 5e). However, β-actin mRNA is known to be transported to distal sites and leading edges within cells [37,38]. Our flow cytometry purification approach necessarily shed distal myelin processes from isolated ODCs (Figure 1b). Accordingly, immunostaining of spinal cord sections showed increased presence of β-actin in myelin structures and ODC processes at disease onset compared to controls (Figure 5e). To investigate other changes in cytoskeleton gene expression, we then analyzed the major component of ODC cytoskeleton, β-tubulin, for which distal mRNA transport has not been described. Our analysis indeed revealed significant upregulation of β-tubulin expression in mature ODCs at clinical onset of EAE compared to control naïve mice (Figure 5f). Altogether, *in vivo* and *ex vivo* analysis of oLucR mice indicates that CNS inflammation induces drastic changes in cytoskeletal genes within the ODC population.

## Discussion

ODCs were originally thought to provide mere structural requirements for saltatory signal transduction of neurons. Only in recent years, they found increasing recognition as fundamental supporters of neuronal metabolism and survival and have thus obtained attention for their active role in many neurodegenerative pathologies [2,3,10]. Mature ODC death and demyelination directly promote the development and accumulation of clinical disabilities within demyelinating diseases such as MS, leukodystrophies, and respective animal models [11,32]. Following such pathological processes, stem cells and OPCs enable a functional healing process through remyelination of insulted CNS areas. The current paradigm holds that during such insults and subsequent remyelination, postmitotic ODCs remain passive victims of damage [16]. In contrast to this traditional viewpoint, different studies support the hypothesis that mature ODCs actively respond to parenchymal stress [39] and maintain some degree of structural plasticity potentially enabling not only ODC survival during neuroinflammation but also complex dynamic adaptation upon environmental cues [21-25]. Unfortunately, little is known about the *in vivo* response and potential functional plasticity of mature ODCs upon demyelinating insults. Underlying the complex cytoarchitecture of ODCs, β-actin and β-tubulin are necessary candidates as main players in ODC and myelin dynamics. Even though both have been traditionally considered housekeeping genes, their transcriptional control can be substantially affected during cell proliferation, activation, differentiation, membrane fragmentation, and in the context of different pathological conditions [33,40-42]. We therefore chose the β-actin promoter in combination with luciferase as a readout to investigate the fine *in vivo* dynamics of mature ODCs. Given its low background, high quantum yield, and good tissue penetration, the luciferase reporter is widely used in macroscopic *in vivo* measurements [43]. In our oLucR mouse model, luciferase expression is controlled by the β-actin promoter and restricted to MOG-expressing ODCs by a Cre/loxP system. Luciferase activity in these mice increased during CNS development followed by a stable, reproducible signal intensity in adult animals, as expected from the known sequence of myelination events [31]. Cre/loxP recombination was highly effective and specific for mature, PLP^+^MOG^+^NG2^negative^ ODCs. Thus, oLucR mice allowed us to follow *in vivo* bioluminescence changes specifically from the terminally differentiated ODC population.

In this study, different experimental insults to myelin and ODCs led to a consistent augmentation of ODC-specific bioluminescence in oLucR mice. First, in the oDTR model [11], widespread ODC death through DTx administration in oLucR/oDTR mice transiently albeit consistently increased *in vivo* bioluminescence. Second, a strong rise in luciferase signal was observed after autoimmune inflammatory myelin and ODC damage, correlating with the clinical disease onset of EAE in oLucR mice. Such variations in luciferase levels appeared intrinsic to the demyelinating CNS as *ex vivo* and *in vitro* analysis of freshly cut CNS slices and CNS protein extracts confirmed these *in vivo* results. Altogether, despite the possibility of signal loss due to ODC death and reduction in ODC numbers, demyelinating conditions led to a consistent increase in the ODC-specific luciferase reporter signal. The post-lesion timing of such bioluminescence changes clearly rules out any possible signal contribution from recruited/differentiated OPCs. PT adjuvant treatment alone, known to increase BBB permeability, resulted in a transient and low signal augmentation, suggesting that enhanced luciferin access to the CNS can account only for a small proportion of the observed fluctuations. We did not formally exclude a direct ‘activatory’ effect of PT on ODCs [44], but this seems unlikely due to the delayed occurrence of enhanced *in vivo* bioluminescence in the EAE model. Nonetheless, the relatively fast decline of *in vivo* bioluminescence following the rapid increases in luciferase emission at clinical onset of EAE or during DTx administration in oLucR/DTR mice remains somewhat puzzling. In DTx-injected oLucR/oDTR mice, the drastic drop in bioluminescence can be easily ascribed to the massive ODC degeneration observed 1 week post DTx administration [11]. However, death of luciferase^+^ ODCs alone cannot explain the bioluminescence decrease following the emission peak observed during EAE, as ODC death in such paradigm is neither massive nor sharply-timed. Rather, such emission kinetics might indicate that mature ODC ‘activation’ represents a transient process which closely mirrors the strong, destructive inflammation observed in the early phases of EAE. Also, increased tissue hypoxia in the inflamed spinal cord [45,46] could directly contribute to the decrease in bioluminescence following disease onset, as luciferase activity is highly dependent on the amount of oxygen available [47]. Overall, the consistent increase of *in vivo* and *in vitro* bioluminescence upon immune- or toxin-mediated demyelination/ODC damage suggests that luciferase^+^ mature ODCs exhibit some kind of stress-induced acute activation of the cytoskeleton encoding genes within and surrounding experimental demyelinating lesions.

Accordingly, total β-actin expression was higher in the CNS of EAE-induced oLucR and DTx-treated oLucR/oDTR mice than in control mice. We have previously shown that in the oDTR model, no lymphocyte infiltration, OPC recruitment, microglia or astroglia activation can be observed 1 week after induction of demyelination [11]. Therefore, in the DTx-treated oLucR/oDTR mouse model, the early increase in actin transcription can be ascribed to a transient mobilization of mature luciferase^+^ ODCs, which were not killed by DTx. Surviving mature ODCs in the oDTR model are generally located in close proximity to damaged cells and within demyelinating CNS areas [11]. ODCs could thus sense pathological changes of dying neighboring ODCs through soluble mediators or via gap junctions within the glial syncytium [48]. However, in oLucR mice at clinical onset of EAE, the observed increase in CNS β-actin expression is likely affected by inflammatory processes such as leukocyte infiltration and glial activation. We thus performed immunostaining of myelin and β-actin in white matter areas at onset of EAE and observed stronger expression of β-actin in ODC processes and attached myelin sheaths. Additionally, we repeated RNA expression analysis on purified mature ODCs through FACS-sorting of EYFP^+^ ODCs but found no difference in β-actin expression upon onset of clinical EAE in sorted ODCs. This apparent conundrum could result from our technical approach of cell purification, as our RT-PCR data can only provide information on mRNA content of the ODC cell body, but not on distally transported mRNAs, which are highly enriched in myelin (that is, *MBP* mRNA [49,50]). Such mechanism of distal transport and translation has been indeed extensively described for β-actin mRNA [37,38] and allows higher polymerization rate of microfilaments and increased cell motility in the leading edge of dynamic cellular structures. It is thus likely that mature ODCs enhance β-actin expression and mRNA distal transport *in vivo* upon demyelinating stress. Different from β-actin mRNA particles, β-tubulin mRNA is not transported to distal cellular compartments, as microtubules are very rare in thin villi of mature cells and leading edges of myelinating progenitors compared to microfilaments [51]. Through our expression analysis on purified MOG^+^ cells, we could thus show for the first time that β-tubulin is significantly increased in mature ODC cell bodies at clinical onset of CNS inflammation. Altogether, demyelination/ODC damage leads to upregulation of the main component of the cytoskeleton, β-tubulin, and hints toward an enhanced distal distribution of upregulated β-actin mRNA in activated mature ODCs.

## Conclusions

Our study addresses a long-standing issue on the biology of mature ODCs within demyelinating CNS: the current perception of postmitotic ODCs as simple passive victims of insults to the myelin. This notion is put under discussion by studies describing different degrees of structural plasticity of mature ODCs in different *in vivo* and *in vitro* experimental scenarios and the presence of significant mechanisms of myelin remodeling existing throughout adulthood [5,18-26,52]. Accordingly, the oLucR model allowed us to 1) indirectly show through the activity of a β-actin-driven reporter gene and 2) directly prove through immunohistochemistry and expression analysis of damaged CNS/sorted cells, that mature ODCs experience a dynamic *in vivo* activation of the cytoskeleton possibly leading to reorganization of myelin sub-structures. Our observations thus suggest that mature ODCs can actively respond to various demyelinating CNS insults, increase their cytoskeletal plasticity *in vivo* upon different types of parenchymal stress, and may thus constitute potential targets for therapies aimed at supporting neuroprotection/remyelination.

## Abbreviations

CNS: central nervous system
DTx: diphtheria toxin
EAE: experimental autoimmune encephalomyelitis
i.p.: intraperitoneal
MS: multiple sclerosis
ODC: oligodendrocyte
OPC: oligodendrocyte precursor
PT: pertussis toxin
